# Evaluation of Antimicrobial Resistance Patterns of *Pseudomonas aeruginosa* Strains Isolated among COVID-19 Patients in Brazil Typed by Fourier-Transform Infrared Spectroscopy

**DOI:** 10.3390/life14091079

**Published:** 2024-08-29

**Authors:** Paula Araujo de Souza, Milena Cristina Silva dos Santos, Rebeca Vitória da Silva Lage de Miranda, Luciana Veloso da Costa, Raphael Paiva Paschoal da Silva, Catia Aparecida Chaia de Miranda, Ana Paula Roque da Silva, Stephen James Forsythe, Maria Helena Simões Villas Bôas, Marcelo Luiz Lima Brandão

**Affiliations:** 1 Laboratory of Microbiology of Food and Sanitizes, INCQS/Fiocruz, Rio de Janeiro 21040-900, Brazil; maria.villas@fiocruz.br; 2 Laboratory of Microbiological Control, Bio-Manguinhos/Fiocruz, Rio de Janeiro 21040-900, Brazil; milena.santos@bio.fiocruz.br (M.C.S.d.S.); rebeca.lage@bio.fiocruz.br (R.V.d.S.L.d.M.); luciana.costa@bio.fiocruz.br (L.V.d.C.); marcelo.brandao@bio.fiocruz.br (M.L.L.B.); 3 Hospital de Força Aérea do Galeão, Força Área Brasileira, Rio de Janeiro 21941-353, Brazil; raphaelpasc@gmail.com; 4 Interdisciplinary Medical Research Laboratory, IOC/Fiocruz, Rio de Janeiro 21040-900, Brazil; catia.chaia@ioc.fiocruz.br; 5 Analytical Indicators and Data Systems Section, Bio-Manguinhos/Fiocruz, Rio de Janeiro 21040-900, Brazil; ana.roque@bio.fiocruz.br; 6 Foodmicrobe.com Ltd., Adams Hill, Keyworth, Nottingham NG12 5GY, UK; sforsythe4j@gmail.com

**Keywords:** *P. aeruginosa*, COVID-19, multidrug-resistant

## Abstract

This study aimed to characterize *Pseudomonas aeruginosa* strains isolated from hospitalized patients during the COVID-19 pandemic. This was achieved using phenotypic and molecular techniques, including their antimicrobial resistance profile and biofilm formation. Eighteen strains were isolated from a hospital in Rio de Janeiro, Brazil, and identified by VITEK^®^2, MALDI-TOF/MS (VITEK MS^®^ and MALDI Biotyper^®^), and 16S rRNA sequencing. Fourier-transform infrared (FTIR) spectroscopy, antimicrobial susceptibility testing, and biofilm formation and disinfectant tolerance tests were applied to evaluate the virulence characteristics of the strains. VITEK^®^2 (≥99%), VITEK MS^®^ (≥82.7%), and MALDI Biotyper^®^ (score ≥ 2.01) accurately identified the *P. aeruginosa* strains, but 16S rRNA sequencing did not differentiate the species *P. aeruginosa* from *P. paraeruginosa*. FTIR typing identified three different clusters, but no correlation between the phenotypical or antimicrobial susceptibility testing patterns was found. Most strains exhibited resistance to various antimicrobials. The exceptions were sensitivity to amikacin and norfloxacin, and consequently, these could be considered potential treatment options. Most strains (n = 15, 83.3%) produced biofilms on polystyrene. Sodium hypochlorite treatment (0.5%/15 min) was shown to be the most effective disinfectant for biofilm elimination. *P. aeruginosa* biofilm formation and tolerance to disinfectants demonstrate the need for effective cleaning protocols to eliminate contamination by this organism in the hospital environment and medical equipment.

## 1. Introduction

*Pseudomonas aeruginosa* is a Gram-negative pathogen commonly associated with healthcare-associated infections (HAIs), causing infections, especially in immunocompromised patients [1]. It is mainly associated with respiratory tract infections, as well as ventilator-associated pneumonia (VAP), meningoencephalitis, and sepsis [2]. Due to its remarkable capacity to resist antibiotics, the eradication of this pathogen has become a challenge. *P. aeruginosa* is intrinsically resistant to several antimicrobials, such as kanamycin and neomycin [3]. It has adaptive resistance, which includes biofilm-mediated resistance and the acquisition of resistance genes through horizontal gene transfer [4]. The quick identification of pathogens in the routine of the microbiology laboratories would speed up the procedures of infection control that contribute to the elimination of *P. aeruginosa* in the hospital environment [5]. Fourier-transform infrared (FTIR) spectroscopy (Bruker Optics-Daltonics, Bremen, Germany), performed with IR Biotyper, is a method that aims to type microorganisms within 3 h [6]. The technique generates spectra based on the absorption of infrared light by the different cell chemical components, providing biochemical fingerprints of the bacterial cells [5]. Since the cell wall composition, membrane structure, and intracellular components vary between strains, each isolate will exhibit a unique FTIR spectrum [7]. The FTIR spectra generated can be compared so that strains can be differentiated and profiled according to their spectral signatures [7]. This technique has been used for the surveillance of several nosocomial Gram-negative rods, including *P. aeruginosa* [5,6,8].

Previous studies have reported the prevalence of 12% *P. aeruginosa* in co-infections with severe acute respiratory syndrome coronavirus 2 (SARS-CoV-2). Furthermore, hospital outbreaks caused by this pathogen have been reported around the world [9,10]. The ability to form a biofilm on medical devices used in endotracheal intubation and mechanical ventilation is likely to be one of the main reasons for the persistence of *P. aeruginosa* [11]. An increase in antibiotic prescriptions was also observed during the COVID-19 pandemic. This could result in the emergence of multidrug-resistant (MDR) *P. aeruginosa* strains and make therapeutic management more difficult [12]. It is plausible that *P. aeruginosa* modifies its virulence factors during co-infection with SARS-CoV-2, leading to greater antimicrobial resistance and biofilm formation [13].

This study aimed to characterize *P. aeruginosa* strains isolated from hospitalized patients during the COVID-19 pandemic using phenotypic and molecular techniques and evaluate their antimicrobial resistance profile and biofilm formation and its tolerance to disinfectants.

## 2. Materials and Methods

### 2.1. Bacterial Strains and Culture Conditions

Eighteen strains identified as *P. aeruginosa* using VITEK^®^2 (bioMérieux, Craponne, France), with a confidence of > 99%, were isolated between July 2021 to March 2022 from a hospital located in Rio de Janeiro State, Brazil. The strains were isolated from tracheal secretion (n = 9), urine (n = 6), rectal swab (n = 2), and oral swab (n = 1) (Table 1). The strains were isolated from patients with COVID-19 and non-COVID-19 co-infection, and from surveillance samples collected from the patients by the Hospital Infection Prevention and Control Committee (HIPCC).

*P. aeruginosa* ATCC 27,853 was used as the control culture for specific tests.

Stock cultures were prepared and maintained at −70 °C in Difco™ Skim Milk 10% (BD Biosciences, Le Pont de Claix, Auvérnia-Ródano-Alpes, France), containing 30% glycerol (Merck, Darmstadt, Germany). The inocula were prepared by transferring one loopful into 3 mL fresh brain-heart infusion broth (BHI) (Merck, Darmstadt, Germany) and incubating at 37 °C for 24 h. For daily use, cultures were maintained at 5 °C on Tryptic Soy Agar (TSA) (BioCen do Brasil, São Paulo, Brazil). All strains were deposited at the Coleção de Bactérias do Ambiente e Saúde (CBAS) hosted at Fundação Oswaldo Cruz (Fiocruz), Rio de Janeiro, Brazil (https://portal.fiocruz.br/, acessed on 15 August 2024). CBAS is affiliated with the World Federation for Culture Collections (WFCC) and registered as the World Data Centre for Microorganisms (WDCM) 958 (https://ccinfo.wdcm.org/details?regnum=958, acessed on 15 August 2024).

### 2.2. Identification by Matrix-Assisted Laser Desorption Ionization Time-of-Flight Mass Spectrometry Proteomic Characterization

The isolates were grown on Sheep Blood Agar (SBA) (BioCen do Brasil, São Paulo, Brazil) at 32.5 °C for 48 h. All strains were identified using MALDI Biotyper^®^ (Bruker Daltonics, Bremen, Germany) and VITEK MS^®^ systems (bioMérieux, Craponne, Lyon, France) according to the manufacturer’s instructions. For VITEK^®^ MS, using the SARAMIS Premium (version 4.0.0.14) program, the strain was considered identified when >75% similarity was obtained. For the MALDI Biotyper^®^, the MBT Compass Library Revision K (2022) was used and the confidence range of 0.00–1.69 meant that no identification was possible for the microorganism, at 1.70–1.99 the identification was of low confidence at the species level, and at 2.00–3.00 the identification was of high confidence at the species level. The strains not identified to species level by both MALDI-TOF MS equipment were subjected to complete 16S rRNA gene sequencing described below.

### 2.3. 16S rRNA Gene Sequencing Analysis

The isolates were identified by 16S rRNA gene Sanger sequencing analysis using the MicroSEQ™ Full Gene 16S rDNA kit (Thermo Fisher Scientific, Waltham, MA, USA), according to the manufacturer’s instructions. The sequences were processed using DNA Star LaserGene SeqMan software v. 7.0.0, and identification results were obtained from the website https://www.ezbiocloud.net/ (Database Update: 7 July 2021, accessed on 23 February 2023). Only species that presented an identification ≥ 98.7% were considered valid [14]. All sequences were deposited at https://www.ncbi.nlm.nih.gov/, acessed on 15 August 2024, and the access numbers are provided in Table 2. The sequences of the strains: *P. aeruginosa* JCM5962 (access number BAMA01000316), *P. paraeruginosa* PA7 (access number ON359917), *P. otitidis* MCC10330 (access number AY953147), *P. lalkuanensis* PE08 (access number MF943158), and *Luteimonas marina* FR1330 (access number EU295459), were included in the phylogenetic relationship of these strains. A phylogenetic tree based on multiple alignments of nearly complete 16S rRNA gene sequences was constructed using the neighbor-joining and the ClustalW algorithm with the software MEGA 11 by employing the Tamura two-parameter model with branch support based on 1000 bootstrap [15].

### 2.4. Typing by FTIR Spectroscopy Analysis

Isolates were cultured on TSA at 37 ± 1 °C for 24 h. Colonies were selected and 1 μL of the colony was mixed well in a suspension vial containing 70% ethanol (50 μL), followed by the addition of 50 μL distilled water and mixing. Three spots (15 μL each) were loaded onto a 96-well silicon microplate. The completely dried plate was mounted on the IR Biotyper^®^ spectrometer (Bruker Optics-Daltonics, Bremen, Germany). Test standards (IRTS1 and IRTS2), spotted in duplicate, were also included. A dendrogram was created with the raw data to cluster the separation spectrum. The cut-off value on the dendrogram was automatically calculated using OPUS v.7.5 software (Bruker Optics-Daltonics GmbH, Bremen, Germany). The distance matrix based on the average Euclidean distance and linkage algorithm reflects the separation distances of all strains.

### 2.5. Antimicrobial Susceptibility Profile

*P. aeruginosa* isolates were evaluated for susceptibility to antimicrobial agents using the disk diffusion agar method, following the criteria of the Clinical and Laboratory Standards Institute (CLSI) (2020) [16]. Antimicrobials tested were sulfamethoxazole-trimethoprim (SXT), meropenem (MEM), ceftazidime (CAZ), cefoxitin (FOX), cefepime (CEF), ceftriaxone (CRO), cefuroxime axetil (CXM), piperacillin-tazobactam (TZP), amikacin (AMI), gentamicin (GEN), ciprofloxacin (CIP), norfloxacin (NOR), and imipenem (IPM), and interpreted following the CLSI [14]. The strains were classified as MDR, extensively drug-resistant (XDR), or pandrug-resistant (PDR) according to the criteria proposed by Magiorakos et al. [17]. The author characterizes MDR as non-susceptible to ≥1 agent in ≥3 antimicrobial classes, XDR as non-susceptible to ≥1 agent in all but ≤2 categories, and PDR as non-susceptible to all antimicrobial agents in all antimicrobial classes [17].

### 2.6. Biofilm Formation and Its Tolerance to Disinfectants

The strains were tested for biofilm formation in polystyrene surfaces as described by Vasconcellos et al. [18]. The strains were grown on tryptic agar soy (TSA) at 37 °C for 24 h. Colonies were selected and cultured on 3 mL of BHI (Merck, Darmstadt, Germany) at 37 °C for 24 h. A total of 200 µL of the culture was inoculated in triplicate on a polystyrene surface in 96 well plates (Falcon^®^, Elizabeth, NJ, USA) and incubated at 22.5 ± 2.5 °C and 37 ± 2 °C for 48 h. The plates were washed five times with distilled water and kept at room temperature until completely dry. An aliquot (200 µL) of 0.41% crystal violet solution (Merck, Darmstadt, Germany) was added to each well and the microplates were kept at room temperature for 45 min. The wells were aspirated, and the microplates were washed five times with water and kept at room temperature until completely dry. Then, 200 µL of 96% absolute ethanol (Merck, Darmstadt, Germany) was added to each well and the microplates were kept under agitation. After 10 min, 150 µL of the contents of each well was transferred to new microplates, and the optical density (O.D.) was read in a spectrophotometer (bioMérieux, Reader 270, Craponne, France).

The assay was repeated in triplicate in three separate experiments for each strain. *P. aeruginosa* ATCC 27,853 was used as positive control. Strains were categorized based on the cut-off optical density (ODC) compared with OD of the negative control: non-adherent (−) OD ≤ ODC, weakly adherent (+) ODC < OD ≤ 2 × ODC, moderately adherent (++) 2 × ODC < OD ≤ 4 × ODC, or strongly adherent (+++) 4 × ODC < OD [19]. The final categorization was the medium of the nine results. 

The strains classified as moderately or strongly adherent were selected to evaluate their tolerance to the following disinfectants: alcohol 70% for 5 min (Merck, Darmstadt, Germany), sodium hypochlorite 0.1% and 0.5% for 15 min (Brasquímica, Belo Horizonte, Brazil), disinfectant based on synergistic association between ammonium quaternary 5th generation and stabilized polymeric biguanide/10 min (Mirax BG diluted 1:200, Hortolândia, Brazil), and peracetic acid 0.5%/10 min (Divosan Forte VT6, Diversey^®^, Peróxidos do Brasil Ltd.a, Curitiba, Brazil). Differences in the degree of biofilm and its tolerance to disinfectants were examined by Wilcoxon signed ranks test using software R Core Team v. 4.2.0 (Vienna, Austria). *p*-values < 0.05 were considered significant.

## 3. Results

### 3.1. Identification and Typing

**Figure 1:**
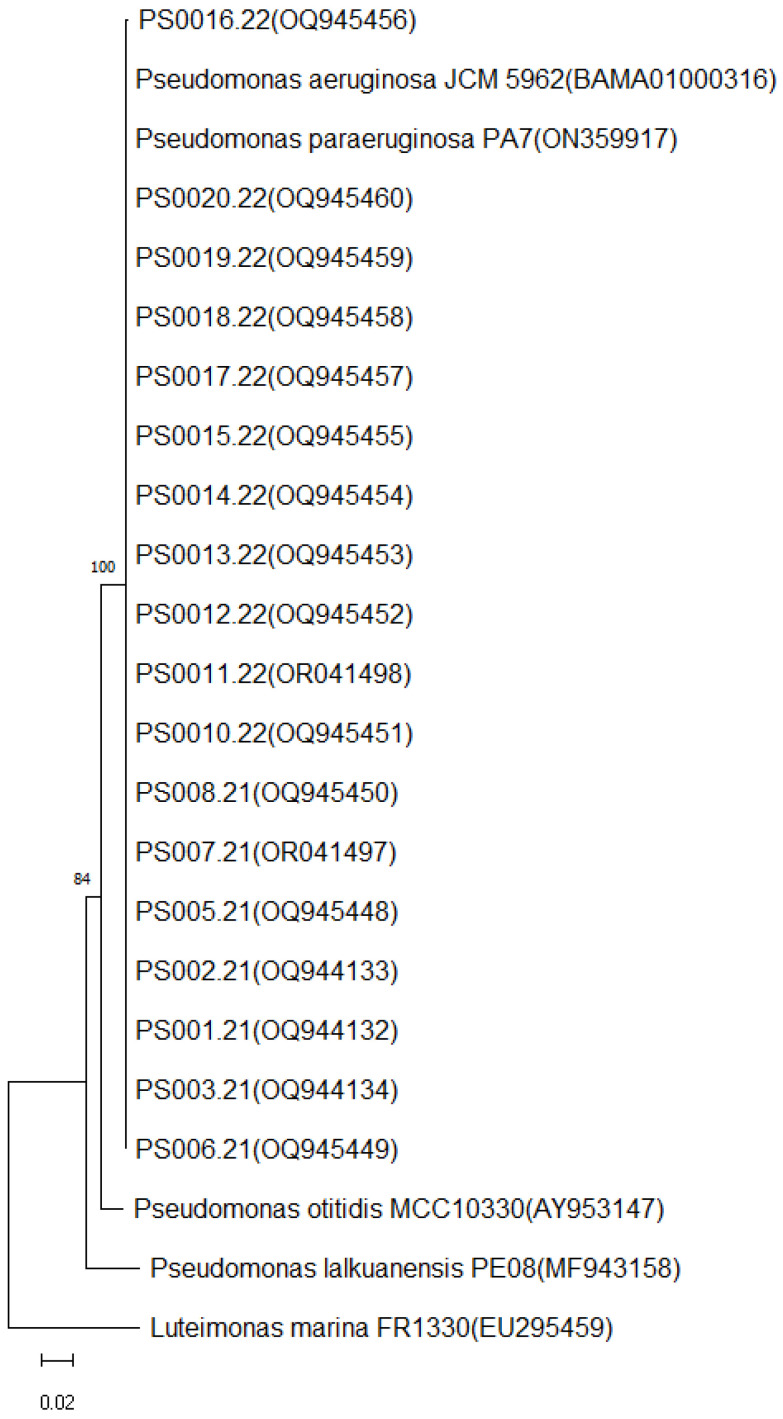
Neighbor-joining tree based on partial 16S rRNA gene sequences (549 bp) showing the phylogenetic position of the strains evaluated in the present study (n = 18). The numbers at the nodes indicate the percentage of 1000 bootstrap replicates; only values > 50% are shown. *Luteimonas marina* FR1330 was used as an outgroup. The scale bar represents 0.02 substitutions per nucleotide position. GenBank accession number is given.

VITEK MS^®^ and MALDI Biotyper^®^ identified all strains as *P. aeruginosa* with ≥82.7% confidence and score ≥ 2.00, respectively (Table 1). According to 16S rRNA gene sequencing analysis, the strains were identified as *P. aeruginosa* with *P. paraeruginosa* as a possibility (Table 2). The neighbor-joining tree clustered the strains together (Figure 1).

**Figure 2:**
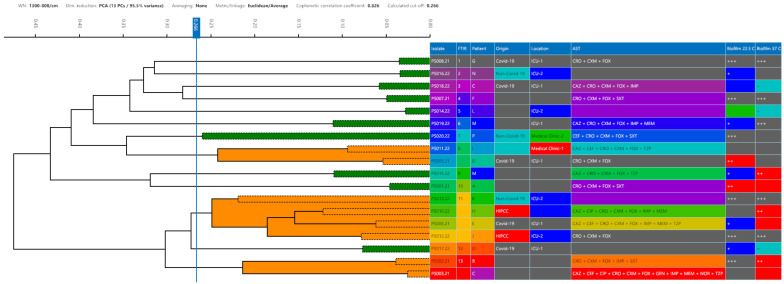
Dendrograms obtained by clustering FTIR spectra for *P. aeruginosa* strains (n = 18). The vertical dashed lines represent the cut-off value. Green spectra indicate that the strain was a singleton, and orange spectra indicate a cluster formation. Different colors are used to differentiate the information regarding patient identification (A–G). Origin (COVID-19 or non-COVID-19 co-infection), antimicrobial susceptibility test (AST) antimicrobials that presented resistance (SXT: sulfamethoxazole-trimethoprim, MEM: meropenem, CAZ: ceftazidime, FOX: cefoxitin, CEF: cefepime, CRO: ceftriaxone, CXM: cefuroxime axetil, TZP: piperacillin-tazobactam, AMI: amikacin, GEN: gentamicin, CIP: ciprofloxacin, NOR: norfloxacin, IPM: imipenem), biofilm formation on polystyrene surface at 22.5 and 37 °C (- = non-adherent; + = weakly adherent; ++ = moderately adherent; and +++ = strongly adherent).

The typing of the 18 isolates obtained from FTIT resulted in 13 different profiles with a 0.266 cut-off (Figure 2). The FTIR profile 11 formed the largest cluster with four strains (PS006, PS010, PS012, and PS013), followed by profiles 8 (PS005 and PS011) and 13 (PS002 and PS003), which clustered two strains each.

### 3.2. Evaluation of Antimicrobial Susceptibility Profile

The antimicrobial susceptibility profiles are presented in Figure 2 and Table 3. All the strains were resistant to CXM, CRO, and FOX but susceptible to AMI. According to EUCAST [3], the CRO resistance phenotype is already expected in *P. aeruginosa* strains, and this fact was observed in this study as predictable. Strain PS003 was the only strain resistant to NOR. Six (33.3%) strains were resistant to SXT, five (27.8%) were resistant to TZP, and eight (44.4%) were intermediate resistant. Eight (44.4%) strains showed intermediate resistance to CAZ and seven (38.9%) were resistant. Two (11.1%) strains were intermediate resistant to MEM, while four (22.2%) were resistant. Regarding the drug CIP, six (33.3%) strains were intermediate resistant and three (16.7%) were resistant. Twelve (66.6%) strains were classified as CEF intermediate resistance, and three (16.7%) were resistant. Seven (38.9%) strains were intermediate resistant to IPM and six (33.3%) were as resistant. The strains PS003 and PS006 were, respectively, resistant and intermediate resistant to GEN, while the remaining strains were susceptible. Fourteen (77.8%) strains (PS001, PS002, PS003, PS006, PS007, PS010, PS011, PS012, PS013, PS014, PS015, PS018, PS019 and PS020) were resistant to ≥5 antimicrobials tested and were classified as MDR according to Magiorakos et al. [17] (Table 3).

Detailed information on the percentage of resistance found for each antimicrobial can be found in Supplementary Table S1.

### 3.3. Evaluation of Biofilm Formation and Its Tolerance to Disinfectants

**Figure 3:**
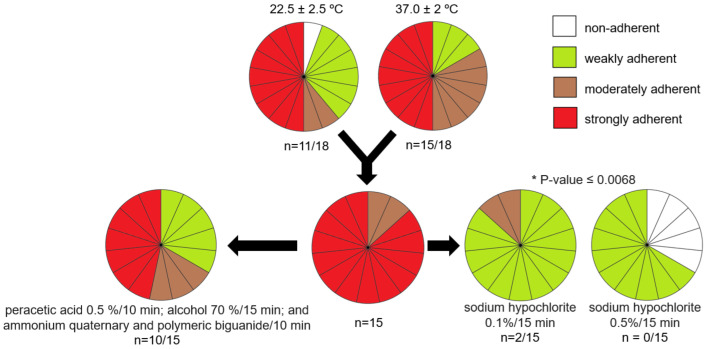
Biofilm formation of *P. aeruginosa* strains (n = 18) and assessment of biofilm sensitivity to disinfectants. Color legend: white: non-adherent; green: weak adherent; brown: moderate adherent; red: strong adherent. Significant *p*-values are presented.

*P. aeruginosa* biofilm formation is presented in Figure 3. No significant statistical difference between the temperatures of incubation (22.5 ± 2.5 °C and 37 ± 2 °C) was observed (*p* = 0.18). At both temperatures, 50.0% (n = 9) of the strains formed strongly adherent biofilm. At 22.5 °C ± 2.5 °C, 11.1% (n = 2) of the strains were classified as moderate biofilm producers, 33.3% (n = 6) of strains produced weakly adherent biofilms and only one strain did not form biofilm. At 37 °C ± 2 °C, 33.3% (n = 6) of the strains were moderately adherent and 16,7% (n = 3) were weakly adherent. 

Overall, 83.3% (n = 15) of the strains were classified as moderately or strongly adherent and were submitted to the biofilm disinfectant tolerance test. Exposure to peracetic acid (0.5%), alcohol (70%), and ammonium quaternary fifth-generation/stabilized polymeric biguanide did not reduce biofilm formation (*p* ≥ 0.0832). Significant statistical difference in biofilm formation was observed only for sodium hypochlorite (0.1% and 0.5%) (*p* = 0.0068); nevertheless, only the higher concentration (0.5%) reduced non-adherence in 22.2% (n = 4) of the strains (Figure 3).

Detailed information on biofilm formation and its tolerance to disinfectants can be found in Supplementary Tables S2 and S3.

## 4. Discussion

*P. aeruginosa* is an opportunistic pathogen that is often related to nosocomial infections, especially in intensive care units (ICUs). It has been responsible for several hospital outbreaks around the world [1]; therefore, its accurate and rapid identification is warranted. Furthermore, accurate bacterial identification is very important for bacterial epidemiology and for the appropriate use of antimicrobials [20]. In the present study, the 16S rRNA gene sequencing was not able to distinguish between *P. aeruginosa* and *P. paraeruginosa*, indicating that these two species have a very close genotypic relationship. These two species aligned together in the phylogenetic tree, showing that this gene is highly conserved between them (Figure 1). Similar results were observed in the study of Rudra et al. [21], which also verified that the 16S rRNA gene sequencing is not a reliable method for *P. aeruginosa* and *P. paraeruginosa* differentiation.

The MALDI-TOF/MS (using both systems) was able to identify all the *P. aeruginosa* strains to the species level (Table 1). MALDI-TOF/MS is a technology used worldwide due to its high accuracy and rapidness while also being cost-effective for bacterial identification [22]. According to Mulet et al. [23], the MALDI-TOF MS proved to maintain high sensitivity and specificity for the early identification of high-risk clones of *P. aeruginosa* in several clinical isolates. Wamer et al. [24] also reported that MALDI-TOF MS is an effective technique for the identification of *P. aeruginosa*.

In the FTIR analysis, the IR 11 profile clustered four strains together which were able to form a moderate or strong biofilm. Three strains (PS010.22, PS012.22, and PS013.22) were isolated from the same location (ICU-2), and two (PS010.22 and PS012.22) of them were isolated during HIPCC monitoring. In addition, biofilms formed by PS010.22 and PS013.22 were tolerant to all disinfectants tested, except for sodium hypochlorite. These results suggest that biofilm formation could have been an important factor in the persistence of this cluster in the hospital. The IR 8 profile clustered two strains (PS005.21 and PS011.21) together. These two strains formed strong biofilms which were tolerant to all disinfectants tested, except for sodium hypochlorite. In the IR 13 profile cluster, which grouped the PS002.21 and PS003.21, it was noted that both strains were isolated from patients with COVID-19 in the ICU-1, and produced strong biofilms which were tolerant to all disinfectants tested, except for sodium hypochlorite. The strains were resistant to ≥5 drugs tested, and 77.78% of the strains were classified as MDR.

Therefore, FTIR was shown to be a useful tool for *P. aeruginosa* typing. In the study of Martak et al. [5], this technique was reported to be a very useful approach for differentiating *P. aeruginosa* clones responsible for hospital outbreaks. These results also corroborate with the previous study where FTIR was used for the surveillance of another nosocomial Gram-negative bacilli, *Stenotrophomonas maltophilia*, where the methodology was able to differentiate strains from clinical and environmental sources [25].

The therapeutic options for *P. aeruginosa* infections are restricted due to its intrinsic and adaptative resistance to antibiotics commonly prescribed [3]. Furthermore, studies indicate an increase of up to 50% in the emergence of MDR *P. aeruginosa* strains during the COVID-19 pandemic, which made therapeutic management even more limited and increased patient mortality [26]. In the present study, all the strains were resistant to cefuroxime axetil, ceftriaxone, and cefoxitin, and 77.78% of the strains were classified as MDR.

For a long time, β-Lactam antibiotics have been a mainstay in the treatment of *P. aeruginosa*; however, several β-Lactam-resistant strains have been reported, which has restricted even more the therapeutic options available [27]. In the present study, the highest rate of resistance was observed in β-Lactam antibiotics, where 83.3% (n = 15) of the strains showed resistance or intermediate resistance to ceftazidime, followed by cefepime with 77.8% (n = 14) and piperacillin-tazobactam with 72.2% (n = 13).

Due to the high emergence of carbapenem-resistant *P. aeruginosa* (CRPA) worldwide, the World Health Organization (WHO) [28] has included CRPA in the list of pathogens of high priority for the research and development of new antibiotics [29]. In the present study, 72.2% (n = 13) of the strains were resistant or intermediate resistant to imipenem and 33.3% (n = 6) were resistant or intermediate resistant to meropenem.

The sulfonamides are considered an alternative for the treatment of *P. aeruginosa* infections resistant to carbapenems [30]. However, due to the ability of *P. aeruginosa* to acquire new resistance via mutation and horizontal gene transfer, resistant strains are being reported [31]. In the present study, 33.3% of the strains were resistant to sulfamethoxazole-trimethoprim. Similar results were observed in the study of Haghi et al. [31], where 77.4% of the *P. aeruginosa* were sulfamethoxazole-trimethoprim resistant.

The aminoglycosides showed favorable activity against *P. aeruginosa* strains since all the strains were susceptible to amikacin and only the strains PS003.21 and PS006.21 were, respectively, resistant and intermediate resistant to gentamicin. Moreover, the fluoroquinolones-class antibiotic, norfloxacin, can also be considered promising treatment options against *P. aeruginosa*; only the strain PS003.21 was resistant. In contrast, 50.0% (n = 9) of the strains were resistant or intermediate resistant to ciprofloxacin. Shiralizadeh et al. [32] also reported an increase in fluoroquinolone resistance rates, such as ciprofloxacin, where 60% of the *P. aeruginosa* were resistant.

The incidence of infections caused by *P. aeruginosa* increased significantly during the COVID-19 pandemic, especially due to its ability to form biofilm in medical devices such as catheters, nebulizers, and humidifiers [9,10,26,33]. In the present study, all the strains were able to adhere and produce biofilms in polystyrene surfaces at 37 °C ± 2 °C and only the strain PS014.22 did not produce biofilms at 22.5 °C ± 2.5 °C. Overall, 83.3% (n = 15) were characterized as moderately or strongly adherent (Figure 3). These data corroborate the study of Patel and Gajjar [34], where 16 *P. aeruginosa* strains were strong or moderate biofilm producers.

The biofilm formation contributes not only to the adherence to insert surfaces but can also mediate resistance to several antibiotics and tolerance to disinfectants, hence contributing to the emergence of MDR strains and making the selection of an appropriate cleaning procedure limited [4,35]. In the disinfectant tolerance test, only sodium hypochlorite was able to reduce the biofilm formation in *P. aeruginosa*, reducing 22.2% of the strains to non-adherence at a concentration of 0.5%. Nevertheless, it cannot be used on corrosion-sensitive surfaces, and, therefore, alternative sanitizers/disinfectants and contact periods need to be considered.

## 5. Conclusions

In conclusion, VITEK MS^®^ and MALDI Biotyper^®^ correctly identified *P. aeruginosa* strains. The 16S rRNA gene sequencing did not differentiate between the species *P. aeruginosa* and *P. paraeruginosa*. FTIR was considered a useful tool for *P. aeruginosa* typing. Most of the strains were resistant or intermediate resistant to the drugs tested. In particular, AMI and NOR were demonstrated to be the best treatment options for *P. aeruginosa*, since almost all the strains were susceptible to these two antimicrobials. Fourteen (77.8%) strains were classified as MDR. Almost all the strains produced biofilms on polystyrene surfaces, and sodium hypochlorite (0.5%) was the most effective disinfectant for its elimination. Nevertheless, it is important that further cleaning protocols are evaluated to eradicate this biofilm-producing organism from different surface materials, and consequently reduce the incidence of infection by *P. aeruginosa*. The small number of strains returned from the hospital is a notable limitation of the study, however, several complementary methodologies were applied aiming to give robustness to the present research.

## Figures and Tables

**Table 1:** Details of *P. aeruginosa* strains (n = 18) isolated in a Brazilian hospital during the COVID-19 pandemic.

Strain ID	Source	Patient ID	Local	Origin	Date	MALDI-TOF MS ^a^	
						VITEK MS^®^ (%) ^b^	MALDI Biotyper^®^ (Score) ^c^
PS001.21	Urine	A	ICU ^d^-1	COVID-19 ^e^	07/12/2021	*P. aeruginosa* (99.9)	*P. aeruginosa* (2.40)
PS002.21	Urine	B	ICU-1	COVID-19	07/14/2021	*P. aeruginosa* (99.9)	*P. aeruginosa* (2.05)
PS003.21	Tracheal secretion	C	ICU-1	COVID-19	07/23/2021	*P. aeruginosa* (84.0)	*P. aeruginosa* (2.27)
PS005.21	Tracheal secretion	D	ICU-1	COVID-19	07/30/2021	*P. aeruginosa* (99.9)	*P. aeruginosa* (2.03)
PS006.21	Tracheal secretion	E	ICU-1	COVID-19	07/28/2021	*P. aeruginosa* (99.9)	*P. aeruginosa* (2.31)
PS007.21	Urine	F	ICU-1	COVID-19	08/06/2021	*P. aeruginosa* (99.9)	*P. aeruginosa* (2.04)
PS008.21	Oral swab	G	ICU-1	COVID-19	08/06/2021	*P. aeruginosa* (95.1)	*P. aeruginosa* (2.03)
PS010.22	Rectal swab	H	ICU-2	HIPCC ^f^	01/27/2022	*P. aeruginosa* (99.9)	*P. aeruginosa* (2.33)
PS011.22	Tracheal secretion	I	Medical Clinic ^g^-1	Non-COVID-19	02/17/2022	*P. aeruginosa* (92.7)	*P. aeruginosa* (2.00)
PS012.22	Rectal swab	J	ICU-2	HIPCC	02/23/2022	*P. aeruginosa* (99.9)	*P. aeruginosa* (2.31)
PS013.22	Urine	K	ICU-2	Non-COVID-19	02/28/2022	*P. aeruginosa* (96.1)	*P. aeruginosa* (2.22)
PS014.22	Urine	L	ICU-2	COVID-19	03/01/2022	*P. aeruginosa* (82.7)	*P. aeruginosa* (2.34)
PS015.22	Tracheal secretion	M	ICU-1	COVID-19	02/24/2022	*P. aeruginosa* (91.6)	*P. aeruginosa* (2.00)
PS016.22	Tracheal secretion	N	ICU-2	Non-COVID-19	03/05/2022	*P. aeruginosa* (92.0)	*P. aeruginosa* (2.04)
PS017.22	Tracheal secretion	O	ICU-1	COVID-19	03/04/2022	*P. aeruginosa* (99.4)	*P. aeruginosa* (2.26)
PS018.22	Tracheal secretion	C	ICU-1	COVID-19	03/09/2022	*P. aeruginosa* (99.9)	*P. aeruginosa* (2.10)
PS019.22	Tracheal secretion	M	ICU-1	COVID-19	03/13/2022	*P. aeruginosa* (99.9)	*P. aeruginosa* (2.01)
PS020.22	Urine	P	Medical Clinic-2	Non-COVID-19	03/17/2022	*P. aeruginosa* (99.9)	*P. aeruginosa* (2.07)

^a^—matrix-assisted laser desorption ionization time-of-flight mass spectrometry performed; ^b^—results analyzed by SARAMIS Premium software v. 4.0.0.14, only strains with a percentage ≥ 75% similarity were considered identified; ^c^—results analyzed by MBT Compass software. Data interpretation was done in line with Bruker’s standard criteria; a species cut-off score value of 2.0 and a genus cut-off score value of 1.7 were applied; ^d^—intensive care unit; ^e^—patient with co-infection with severe acute respiratory syndrome coronavirus 2; ^f^—Hospital Infection Prevention and Control Committee; ^g^—medical clinic.

**Table 2:** 16S rDNA sequencing analysis of *P. aeruginosa* strains (n = 18).

Strain ID	NCBI ^a^ Access Number	Base Pair Length	Identification (%)
PS001/21	OQ944132	1027	*P. aeruginosa* (100)*/P. paraeruginosa* (100)
PS002/21	OQ944133	856	*P. aeruginosa* (100)*/P. paraeruginosa* (100)
PS003/21	OQ944134	858	*P. aeruginosa* (99.30)*/P. paraeruginosa* (99.30)
PS005/21	OQ945448	873	*P. aeruginosa* (100)*/P. paraeruginosa* (100)
PS006/21	OQ945449	928	*P. aeruginosa* (99.78)*/P. paraeruginosa* (99.78)
PS007/21	OR041497	1337	*P. aeruginosa* (100)*/P. paraeruginosa* (100)
PS008/21	OQ945450	849	*P. aeruginosa* (100)*/P. paraeruginosa* (100)
PS010/22	OQ945451	874	*P. aeruginosa* (100)*/P. paraeruginosa* (100)
PS011/22	OR041498	1333	*P. aeruginosa* (100)*/P. paraeruginosa* (100)
PS012/22	OQ945452	873	*P. aeruginosa* (100)*/P. paraeruginosa* (100)
PS013/22	OQ945453	588	*P. aeruginosa* (100)*/P. paraeruginosa* (100)
PS014/22	OQ945454	1016	*P. aeruginosa* (100)*/P. paraeruginosa* (100)
PS015/22	OQ945455	975	*P. aeruginosa* (100)*/P. paraeruginosa* (100)
PS016/22	OQ945456	922	*P. aeruginosa* (99.89)*/P. paraeruginosa* (99.89)
PS017/22	OQ945457	1003	*P. aeruginosa* (99.90)*/P. paraeruginosa* (99.90)
PS018/22	OQ945458	935	*P. aeruginosa* (100)*/P. paraeruginosa* (100)
PS019/22	OQ945459	905	*P. aeruginosa* (100)*/P. paraeruginosa* (100)
PS020/22	OQ945460	1025	*P. aeruginosa* (99.41)*/P. paraeruginosa* (99.41)

^a^—National Center for Biotechnology Information.

**Table 3:** Antimicrobial susceptibility profile of *P. aeruginosa* strains (n = 18) by disk diffusion method.

Strains	SXT	CAZ	CEF	CRO	CXM	FOX	TZP	AMI	GEN	CIP	NOR	MEM	IPM	Magiorakos et al. (2012) Classification [17]
PS001.21	R	I	I	R	R	R	I	S	S	S	S	S	I	MDR
PS002.21	R	I	I	R	R	R	I	S	S	I	S	S	R	MDR
PS003.21	S	R	R	R	R	R	R	S	R	R	R	R	R	MDR
PS005.21	S	I	I	R	R	R	I	S	S	S	S	S	I	N-MDR
PS006.21	S	R	R	R	R	R	R	S	I	I	S	R	R	MDR
PS007.21	R	I	I	R	R	R	I	S	S	I	S	S	I	MDR
PS008.21	S	S	S	R	R	R	S	S	S	S	S	S	S	N-MDR
PS010.22	S	R	I	R	R	R	S	S	S	R	S	R	R	MDR
PS011.22	S	R	R	R	R	R	R	S	S	S	S	S	S	MDR
PS012.22	S	I	I	R	R	R	R	S	S	R	S	I	I	MDR
PS013.22	R	I	I	R	R	R	I	S	S	I	S	S	I	MDR
PS014.22	R	I	I	R	R	R	I	S	S	I	S	S	I	MDR
PS015.22	S	R	I	R	R	R	R	S	S	S	S	S	S	MDR
PS016.22	S	S	S	R	R	R	S	S	S	S	S	S	S	N-MDR
PS017.22	S	S	S	R	R	R	S	S	S	S	S	S	S	N-MDR
PS018.22	S	R	I	R	R	R	I	S	S	S	S	I	R	MDR
PS019.22	S	R	I	R	R	R	S	S	S	S	S	R	R	MDR
PS020.22	R	I	I	R	R	R	I	S	S	I	S	S	I	MDR

R—resistant, I—intermediate resistant, S—sensitive, SXT—sulfamethoxazole-trimethoprim, MEM—meropenem, CAZ—ceftazidime, FOX—cefoxitin, CEF—cefepime, CRO—ceftriaxone, CXM—cefuroxime axetil, TZP—piperacillin-tazobactam, AMI—amikacin, GEN—gentamicin, CIP—ciprofloxacin, NOR—norfloxacin, IPM—imipenem, MDR—multidrug-resistant, N-MDR—no-multidrug-resistant.

## Data Availability

The data underlying this article are available in the article.

## Supplementary Materials

The following supporting information can be downloaded at: https://www.mdpi.com/article/10.3390/life14091079/s1, Table S1: Percentages of resistance, intermediary resistance and susceptibility of the *P. aeruginosa* strains (n = 18) to each antibiotic tested; Table S2: Biofilm formation of *P. aeruginosa* strains (n = 18); Table S3: Assessment of *P. aeruginosa* strain (n = 15) biofilm sensitivity to disinfectants.
