# Supplementary material for: Evaluation of Antimicrobial Resistance Patterns of Pseudomonas aeruginosa Strains Isolated among COVID-19 Patients in Brazil Typed by Fourier-Transform Infrared Spectroscopy
Source: Life (Basel). 2024 Aug 29;14(9):1079. doi: 10.3390/life14091079 (PMC11433527; doi:10.3390/life14091079)
Supplement: Supplementary file 1 [file life-14-01079-s001.zip › life-3146343-supplementary.pdf]

Supplementary Table S1. Percentages of resistance, intermediary resistance and susceptibility of the *P. aeruginosa* strains (n=18) to each antibiotic tested.

Antibiotic tested	Result (%)		
	Resistant	Intermediary resistant	Susceptible
SXT	33.3	0	66.7
MEM	22.2	11.1	66.7
CAZ	38.9	44.4	16.7
FOX	100	0	,
CEF	16.7	66.6	16.7
CRO	100	0	0
CXM	100	0	0
TZP	27.8	44.4	44.4
AMI	0	0	100
GEN	5.5	5.5	89.0
CIP	16.7	33.3	50.0
NOR	5.5	0	94.5
IPM	33.3	38.9	27.8

SXT- sulfamethoxazole-trimethoprim, MEM- meropenem, CAZ- ceftazidime, FOX- cefoxitin, CEF- cefepime, CRO- ceftriaxone, CXM- cefuroxime axetil, TZP- pipera-cillin-tazobactam, AMI-amikacin, GEN- gentamicin, CIP- ciprofloxacin, NOR- norfloxacin, IPM- imipenem.

Supplementary Table S2. Biofilm formation of *P. aeruginosa* strains (n=18).

Strains	22.5 ± 2.5ºC	37 ± 2.5ºC
PS001.21	2	2
PS002.21	3	2
PS003.21	3	2
PS005.21	2	3
PS006.21	1	2
PS007.21	3	3
PS008.21	3	3
PS010.22	3	2
PS011.22	3	3
PS012.22	3	3
PS013.22	3	3
PS014.22	0	1
PS015.22	1	2
PS016.22	1	3
PS017.22	1	1
PS018.22	1	1
PS019.22	1	3
PS020.22	3	3

Non-adherent- 0, weakly adherent- 1, moderately adherent- 2, strongly adherent- 3.

Supplementary Table S3. Assessment of *P. aeruginosa* strain (n=15) biofilm sensitivity to disinfectants.

Strains	Alcohol 70%	Peracetic acid 0.5%	Ammonium quaternary and polymeric biguanide	Sodium hypochlorite 0.1%	Sodium hypochlorite 0.5%
PS001.21	2	2	3	2	0
PS002.21	3	3	3	1	1
PS003.21	3	2	2	1	0
PS005.21	3	3	3	1	1
PS006.21	2	1	1	1	1
PS007.21	1	2	2	1	1
PS008.21	3	3	3	2	0
PS010.22	2	3	2	1	1
PS011.22	3	3	3	1	1
PS012.22	1	1	1	1	0
PS013.22	3	3	3	1	0
PS015.22	1	1	1	1	1
PS016.22	1	1	1	1	1
PS019.22	1	1	1	1	1
PS020.22	3	3	3	1	1

Non-adherent- 0, weakly adherent- 1, moderately adherent- 2, strongly adherent- 3.
